# Neurofibromatosis type 1–associated gastric gastrointestinal stromal tumor with PRC2 loss

**DOI:** 10.1007/s12328-026-02362-9

**Published:** 2026-05-23

**Authors:** Masaya Iwamuro, Takehiro Tanaka, Shinsuke Hashida, Duy Hung Ngo, Yusuke Aya, Chihiro Sakaguchi, Yoshiyasu Kono, Hidetaka Yamamoto, Motoyuki Otsuka

**Affiliations:** 1 Department of Gastroenterology and Hepatology, Okayama University Graduate School of Medicine, Dentistry, and Pharmaceutical Sciences, Okayama, 700-8558 Japan; 2 Department of Pathology, Okayama University Graduate School of Medicine, Dentistry, and Pharmaceutical Sciences, Okayama, 700-8558 Japan; 3 Department of Surgery, Okayama City Hospital, Okayama, 700-8557 Japan

**Keywords:** *CDKN2A/B* loss, Gastrointestinal stromal tumor, Neurofibromatosis type 1, PRC2-related epigenetic alteration, Wild-type GIST

## Abstract

Neurofibromatosis type 1 (NF1)-associated gastrointestinal stromal tumor (GIST) is a distinct subtype of wild-type GIST driven by loss of neurofibromin function rather than *KIT* or *PDGFRA* mutations. Although most NF1-associated GISTs exhibit relatively indolent behavior, a subset demonstrates aggressive progression, and the molecular mechanisms underlying this malignant transformation remain unclear. Here, we report a rare case of NF1-associated gastric GIST with polycomb repressive complex 2 (PRC2)-related epigenetic alteration. A 64-year-old man with NF1 was found to have a small submucosal gastric lesion on screening endoscopy. The tumor enlarged rapidly, and laparoscopic local resection was performed. Histopathological examination confirmed a high-risk gastric GIST with positivity for c-*KIT*, DOG-1, and CD34. Immunohistochemistry demonstrated preserved SDHB expression but complete loss of H3K27me3. Comprehensive genomic profiling revealed *CDKN2A* loss, *CDKN2B* loss, *EED* loss, and *MYC* amplification, without pathogenic mutations in *KIT* or *PDGFRA*. Despite surgery and tyrosine kinase inhibitor therapy, the tumor rapidly progressed with peritoneal dissemination and malignant pleural effusion, and the patient died 17 months after surgery. This case highlights a possible association between secondary genomic alterations, PRC2-related epigenetic alteration, and aggressive tumor behavior in NF1-associated GIST. Integrated genomic and epigenomic evaluations may provide important insights into tumor behavior and therapeutic strategies for this rare GIST subtype.

## **Background**

Gastrointestinal stromal tumors (GISTs) are the most common mesenchymal tumors of the gastrointestinal tract and are typically driven by activating mutations in the *KIT* or platelet-derived growth factor receptor alpha (*PDGFRA*) genes. These oncogenic mutations account for approximately 80–85% of sporadic GISTs and constitute the molecular basis for the clinical efficacy of tyrosine kinase inhibitors [1, 2]. In contrast, a distinct subset of GISTs lacks *KIT* and *PDGFRA* mutations and is classified as wild-type GIST. Among these, neurofibromatosis type 1 (NF1)-associated GISTs represent a unique biological entity characterized by loss of neurofibromin function and aberrant activation of the RAS–MAPK signaling pathway [3, 4]. NF1-associated GISTs typically arise in the small intestine, often present as multiple lesions, and are resistant to standard tyrosine kinase inhibitors [3].

Although NF1-associated GISTs are generally considered low- to intermediate-risk tumors with indolent clinical behavior, a subset exhibits aggressive malignant progression. However, the molecular mechanisms underlying this transformation remain unclear. Recent genomic studies have suggested that secondary genetic alterations, including *CDKN2A/B* loss and *MYC* amplification, may contribute to tumor progression in wild-type GISTs [5, 6]. Furthermore, epigenetic dysregulation involving polycomb repressive complex 2 (PRC2), particularly loss of H3K27 trimethylation, has been recognized as a hallmark of malignant peripheral nerve sheath tumors (MPNSTs), which frequently arise in patients with NF1 [7, 8]. However, the role of PRC2-related epigenetic alteration in the malignant progression of NF1-associated GISTs has not been systematically explored.

Here, we report a rare case of high-risk NF1-associated gastric GIST with aggressive clinical behavior and multiple secondary oncogenic alterations, including *CDKN2A/B* loss, *MYC* amplification, and PRC2 alteration accompanied by H3K27me3 loss. This case report provides important insights into the molecular evolution and malignant potential of NF1-associated GISTs.

## Case presentation

**Fig. 1:**
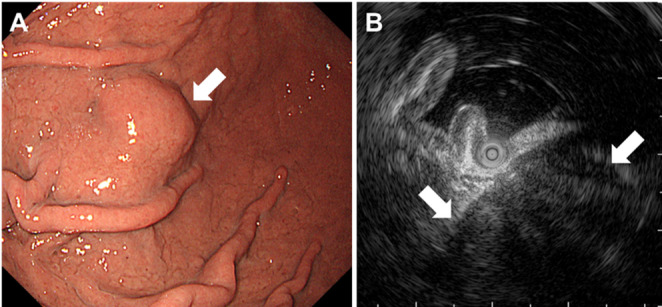
Endoscopic and endoscopic ultrasonographic findings. Esophagogastroduodenoscopy revealed a small submucosal lesion on the greater curvature of the lower gastric body (**A**, arrow). Endoscopic ultrasonography demonstrated a hypoechoic mass continuous with the muscularis propria and predominantly growing extraluminally (**B**, arrows)

**Fig. 2:**
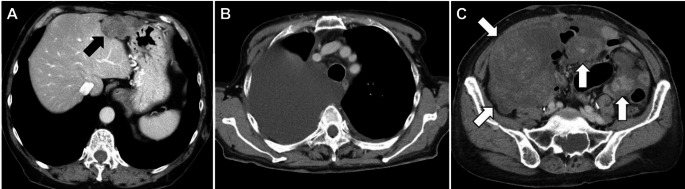
Computed tomography findings during clinical course. Contrast-enhanced computed tomography performed two months after the initial endoscopic examination demonstrated enlargement of an extraluminally growing gastric mass (**A**, arrow). Follow-up CT during disease progression revealed massive right pleural effusion with complete atelectasis of the right lung (**B**). CT simultaneously demonstrated marked progression of peritoneal dissemination (**C**, arrows)

A 64-year-old Japanese man underwent esophagogastroduodenoscopy at a local clinic as part of an annual screening examination. He had previously been diagnosed with NF1 on the basis of multiple café-au-lait spots and cutaneous neurofibromas [9]. He had no gastrointestinal symptoms at presentation and no family history of gastrointestinal malignancy. Esophagogastroduodenoscopy revealed a 10-mm submucosal lesion on the greater curvature of the lower body of the stomach (Fig. 1A, arrow). The patient was referred to a tertiary care center for further evaluation. Endoscopic ultrasonography revealed a hypoechoic mass continuous with the muscularis propria that predominantly exhibited extraluminal growth (Fig. 1B, arrows). The internal echogenicity was slightly heterogeneous. A bite-on-bite biopsy revealed no histological evidence of neoplasia. Two months after the initial diagnosis, follow-up contrast-enhanced computed tomography (CT) revealed progressive enlargement of the extraluminally growing gastric mass (Fig. 2A, arrow). Given the rapid tumor growth and suspicion of malignancy, surgical resection was indicated. As the tumor was localized and no distant metastases were identified on preoperative imaging, laparoscopic wedge resection was selected to achieve complete en bloc resection with adequate margins in a minimally invasive manner while minimizing the risk of tumor rupture. The tumor was resected without rupture. Intraoperatively, several small nodular lesions were observed on the surface of the left hepatic lobe and the peritoneum adjacent to the stomach, which were considered suggestive of peritoneal dissemination. The nodules were excised for diagnostic evaluation.

**Fig. 3:**
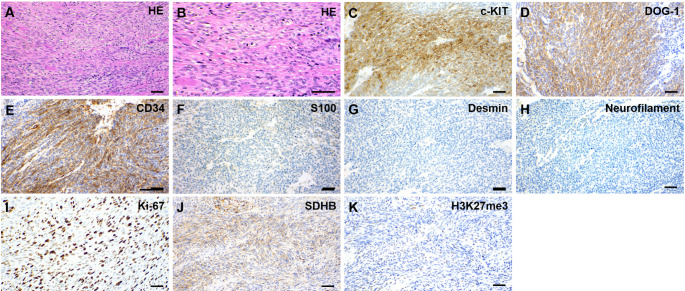
Histopathological and immunohistochemical findings of the tumor. Hematoxylin and eosin staining showed spindle-shaped tumor cells arranged in intersecting fascicles (**A**, **B**). Tumor cells were positive for c-KIT (**C**), DOG-1 (**D**), CD34 (**E**), and Ki-67 (**I**), whereas they were negative for S100 protein (**F**), desmin (**G**), and neurofilament (**H**). Tumor cells also showed preserved SDHB expression (**J**). H3K27me3 immunostaining demonstrated loss of nuclear expression in tumor cells, consistent with PRC2 dysfunction (**K**)

Gross examination of the resected specimen revealed a solid tumor measuring 87 × 64 mm located at the greater curvature of the lower gastric body and extending toward the greater curvature. Histologically, the tumor consisted of spindle-shaped cells arranged in intersecting fascicles (Fig. 3A and B). The tumor cells exhibited elongated nuclei with moderate nuclear atypia and increased cellularity. The mitotic count was 7 per 50 high-power fields. Immunohistochemical analysis showed diffuse positivity for c-KIT (Fig. 3C), DOG-1 (Fig. 3D), and CD34 (Fig. 3E), whereas the tumor was negative for S100 protein (Fig. 3F), desmin (Fig. 3G), and neurofilament (Fig. 3H). The Ki-67 labeling index was approximately 30% (Fig. 3I). Based on these findings, the tumor was diagnosed as a high-risk gastric GIST according to both the Fletcher and Miettinen classifications. Additional immunohistochemical studies demonstrated preserved SDHB expression, indicating SDH-intact GIST (Fig. 3J). However, loss of H3K27me3 expression was observed (Fig. 3K). The primary antibodies used were as follows: c-KIT (polyclonal; Agilent Technologies, Santa Clara, CA, USA), DOG-1 (clone SP31; Roche Diagnostics, Basel, Switzerland), CD34 (clone NU-4A1; Nichirei Biosciences, Tokyo, Japan), Ki-67 (clone MIB-1; Agilent Technologies), S100 protein (polyclonal; Agilent Technologies), desmin (clone D33; Agilent Technologies), neurofilament (clone 2F11; Nichirei Biosciences), SDHB (clone 21A1AE7; abcam, Cambridge, UK), and H3K27me3 (clone C36B11; Cell Signaling Technology, Danvers, MA, USA).

No distant metastases were identified preoperatively, and although peritoneal dissemination was suspected intraoperatively, histopathological evaluation of the nodules revealed no malignancy. Therefore, the tumor was considered non-metastatic at the time of surgery, and complete (R0) resection was achieved. Despite the presence of NF1, the tumor exhibited a typical GIST immunophenotype, and adjuvant therapy with imatinib (400 mg/day) was initiated approximately one month after surgery. The patient remained recurrence-free for approximately 12 months. Thirteen months after surgery, he developed intermittent abdominal pain associated with coughing. Fourteen months after surgery, follow-up CT revealed peritoneal dissemination, and the patient was referred to our institution for further management. Second-line therapy with sunitinib was initiated, and comprehensive cancer genomic profiling was planned.

Comprehensive cancer genomic profiling was performed using the FoundationOne CDx assay (Foundation Medicine, Cambridge, MA, USA) on the previously resected gastric specimen, revealing *CDKN2A* loss, *CDKN2B* loss, *EED* loss, and *MYC* amplification but did not identify pathogenic mutations in *KIT* or *PDGFRA*. In addition, targeted mutational analysis further confirmed the absence of pathogenic variants in the *KIT* gene across exons 8, 9, 11, 13, 14, 17, and 18, and in the *PDGFRA* gene across exons 12, 14, and 18, covering the major hotspot regions of KIT.

Seventeen months after the initial surgery, the patient was admitted to the emergency department with progressive dyspnea. CT revealed massive right pleural effusion with complete atelectasis of the right lung without evidence of pulmonary metastases (Fig. 2B). Concurrently, the peritoneal dissemination showed marked progression (Fig. 2C, arrows). On hospital day 2, right thoracic drainage was performed, yielding reddish turbid pleural fluid that was clinically diagnosed as malignant pleural effusion. Despite supportive care and palliative management, including opioid-based pain control and electrolyte correction, the patient’s general condition progressively deteriorated. On hospital day 33, the patient died of progressive disease. Postmortem findings revealed disseminated nodular lesions on the diaphragm consistent with pleural dissemination.

## Discussion

NF1-associated GISTs represent a biologically distinct subtype of wild-type GIST that differs fundamentally from *KIT*/*PDGFRA*-mutant tumors [3, 4]. These tumors arise as a consequence of neurofibromin deficiency, leading to constitutive activation of RAS signaling and subsequent dysregulation of the downstream MAPK pathway. In contrast to sporadic GISTs, which are primarily driven by activating mutations in *KIT*—most commonly in exon 11, followed by exon 9 and less frequently in exons 13 and 17—NF1-associated GISTs typically lack *KIT* and *PDGFRA* mutations and are therefore classified as wild-type GISTs with limited sensitivity to tyrosine kinase inhibitors. In the present case, comprehensive genomic profiling and targeted mutational analysis covering the major hotspot regions (exons 8, 9, 11, 13, 14, 17, and 18) did not identify any pathogenic KIT mutations, which is consistent with the molecular characteristics of NF1-associated GIST.

Notably, a discrepancy was observed between the immunohistochemical and genomic findings regarding c-KIT. Although the tumor showed diffuse positivity for c-KIT by immunohistochemistry, no pathogenic mutations in the *KIT* gene were identified by comprehensive genomic profiling. This apparent discrepancy is well recognized in NF1-associated GISTs, which typically lack activating mutations in *KIT* or *PDGFRA* but retain expression of KIT protein [3, 4]. In these tumors, oncogenic signaling is primarily driven by loss of neurofibromin function and subsequent activation of the RAS–MAPK pathway rather than by constitutive activation of KIT. Therefore, c-KIT expression in this context likely reflects preserved differentiation toward interstitial cells of Cajal rather than oncogenic driver activity [3, 4]. This distinction may also explain the limited efficacy of tyrosine kinase inhibitors in NF1-associated GISTs.

Clinically, NF1-associated GISTs most commonly develop in the small intestine and are often multifocal [3]. Gastric involvement is relatively uncommon, and solitary large gastric tumors, as observed in the present case, are rare. Although many NF1-associated GISTs demonstrate indolent behavior, high-risk cases with aggressive clinical courses have been reported, suggesting biological heterogeneity within this subgroup. In addition, atypical clinical presentations, such as lymph node metastasis, have been reported [10], although these features do not necessarily correlate with KIT mutation status.

In the present case, the tumor exhibited several molecular features associated with malignant progression. Notably, loss of *CDKN2A* and *CDKN2B* was identified. These genes encode the tumor suppressors p16^INK4A and p15^INK4B, which regulate cell cycle progression through the RB pathway. Loss of *CDKN2A*/*B* is a well-established marker of aggressive behavior in multiple sarcoma subtypes [11] and has been reported as a recurrent secondary alteration in advanced GISTs. This loss likely contributed to uncontrolled cell proliferation, as reflected by the high Ki-67 labeling index. In a large-scale comprehensive genomic profiling study of 144 Japanese patients with advanced GISTs using the National Center for Cancer Genomics and Advanced Therapeutics (C-CAT) database, alterations in cell-cycle–related genes were among the most frequent secondary events, with *CDKN2A* loss reported in 37% and *CDKN2B* loss in 29% of cases [12]. Notably, *CDKN2A/B* loss was observed exclusively in *KIT/PDGFRA*-mutated GISTs, whereas *KIT/PDGFRA*–wild-type tumors harbored alterations in SDH-related genes and other distinct oncogenic events. In this context, the presence of *CDKN2A/B* loss in our *KIT/PDGFRA*–wild-type NF1-associated gastric GIST is an unusual finding and may reflect acquisition of an aggressive, “advanced-type” molecular program beyond the conventional genomic background of wild-type GIST.

In addition, *MYC* amplification was detected. *MYC* is a master transcriptional regulator involved in cell growth, metabolism, and genomic instability [13]. Although *MYC* amplification is relatively uncommon in GISTs, it is associated with high proliferative activity, early recurrence, and poor prognosis in various malignancies. In this case, the combination of *CDKN2A/B* loss and *MYC* amplification suggested a molecular profile consistent with a highly aggressive sarcoma phenotype.

The most distinctive molecular feature of this case was the loss of EED and the complete absence of H3K27me3 expression, indicating dysfunction of the PRC2 complex. PRC2 plays a central role in epigenetic gene silencing through trimethylation of histone H3 at lysine 27 [14, 15]. Loss of PRC2 function and H3K27me3 expression are characteristic hallmarks of MPNST and have emerged as diagnostic markers for these tumors, particularly in the context of NF1. Importantly, genomic profiling was conducted on the resected gastric specimen obtained before the initiation of molecularly targeted therapy. Thus, the identified genomic alterations are considered to reflect the intrinsic molecular features of the tumor rather than treatment-induced changes.

Interestingly, despite an epigenomic profile resembling that of MPNST, the tumor in this case showed unequivocal morphological and immunophenotypic features of GIST, including expression of c-KIT, CD34, and DOG-1, and absence of neural or myogenic markers. This finding suggests that the tumor originated as an NF1-associated GIST but subsequently acquired MPNST-like epigenomic abnormalities during malignant progression. From a biological perspective, this case supports the concept that NF1-associated mesenchymal tumors share common malignant evolutionary pathways regardless of their initial lineage. In particular, PRC2 dysfunction may be associated with aggressive tumor behavior and could represent a potential mechanism of epigenetic reprogramming in NF1-associated tumors. Therapeutically, the molecular profile observed in this case provides an explanation for the limited response to tyrosine kinase inhibitors. NF1-associated GISTs lack *KIT*/*PDGFRA* driver mutations and are therefore intrinsically resistant to imatinib. Furthermore, secondary alterations such as *CDKN2A*/*B* loss, *MYC* amplification, and PRC2-related epigenetic alteration are associated with resistance to targeted therapies and may favor alternative treatment strategies, including CDK4/6 inhibitors, bromodomain and extra-terminal domain inhibitors targeting *MYC*-driven transcription, or epigenetic therapies targeting PRC2-related pathways [16–18].

NF1-associated GISTs typically arise as multiple lesions in the small intestine and often exhibit relatively indolent clinical behavior. In contrast, the present case showed a solitary large gastric tumor with rapid growth and aggressive clinical progression, representing an atypical phenotype. It remains unclear whether this unusual presentation is directly related to the genomic alterations identified in this case. Although no direct evidence currently links specific secondary genomic alterations, such as *CDKN2A/B* loss, *MYC* amplification, or PRC2 dysfunction, to tumor distribution or multiplicity, these alterations may influence tumor behavior and growth patterns. Therefore, this case raises the possibility that additional genomic events contribute to phenotypic heterogeneity in NF1-associated GISTs. Further studies are needed to clarify this relationship.

## Conclusion

This case illustrates a rare but clinically important subtype of NF1-associated gastric GIST characterized by the acquisition of multiple malignant drivers and PRC2-related epigenetic alteration. These findings suggest that epigenomic reprogramming may play a critical role in the malignant evolution of NF1-associated GIST and highlight the need for integrated genomic and epigenomic profiling to better understand tumor behavior and guide therapeutic strategies in this unique patient population.

## Supplementary Information

Below is the link to the electronic supplementary material.
